# Ocular, scrotal and abdominal trauma in a secondary blast injury

**DOI:** 10.1093/jscr/rjad126

**Published:** 2023-03-18

**Authors:** Dennis Machaku, Mkunde Chambo, Mugisha Nkoronko, Mathayo Shadrack, Adnan Sadiq, David Msuya

**Affiliations:** Department of General Surgery, Kilimanjaro Christian Medical Centre, PO Box 3010, Moshi, Kilimanjaro, Tanzania; Kilimanjaro Christian Medical University College, PO Box 3010, Moshi, Tanzania; Department of General Surgery, Kilimanjaro Christian Medical Centre, PO Box 3010, Moshi, Kilimanjaro, Tanzania; Kilimanjaro Christian Medical University College, PO Box 3010, Moshi, Tanzania; Department of General Surgery, Kilimanjaro Christian Medical Centre, PO Box 3010, Moshi, Kilimanjaro, Tanzania; Department of Radiology, Kilimanjaro Christian Medical Centre, PO Box 3010, Moshi, Tanzania; Department of General Surgery, Kilimanjaro Christian Medical Centre, PO Box 3010, Moshi, Kilimanjaro, Tanzania; Kilimanjaro Christian Medical University College, PO Box 3010, Moshi, Tanzania; Kilimanjaro Christian Medical University College, PO Box 3010, Moshi, Tanzania; Department of Radiology, Kilimanjaro Christian Medical Centre, PO Box 3010, Moshi, Tanzania; Department of General Surgery, Kilimanjaro Christian Medical Centre, PO Box 3010, Moshi, Kilimanjaro, Tanzania; Kilimanjaro Christian Medical University College, PO Box 3010, Moshi, Tanzania

**Keywords:** blast injury, surgical debridement, decontamination, trauma triad, case report

## Abstract

Blast injuries are subjected to high morbidity and mortality in the general population. They cognate to single or multiple organ-related injuries that may be life-threatening. The unique injury patterns of blast injuries make treatment therapy complex. An adult male patient presented to our setting with multiple severe deep burn wounds resulting from a dynamite explosion. His computed tomography (CT) scan revealed numerous sharp shards around his body and a ruptured hemiscrotum with exposed testicles. Surgery was immediately done and with a good post-operative outcome. The severity of these injuries escalates in relation to the proximity of the explosions. A CT scan is an imperative diagnostic imaging modality. Treatment involves resuscitation, optimization, excision of non-viable tissues and damage control surgery. Delays in management may have detrimental consequences. Therefore, for physicians to manage the diverse injury manifestations that these patients may present with, they must grasp the pathophysiological patterns of blast injuries.

## INTRODUCTION

Despite increased occurrence in recent years, blast injuries are still uncommon in the general population. As a result of the body being subjected to excessive pressure, they are linked to several injuries. They may have little effect or affect several organ systems [1]. Burns, chest injuries, abdominal injuries, eardrum perforations, genital injuries and damage to the limbs are only a few of the possible ailments. Their presentation to an emergency physician should result in timely identification, evaluation and treatment. This case study intends to describe a patient who arrived at our facility after being injured by a dynamite explosion and suffering multiple burns to his right forearm, penis and scrotum, as well as several bruises on his upper and lower limbs, the anterior aspect of his chest and abdomen, and his left eye. The study will also designate the management strategy and related literature.

## PRESENTATION OF CASE

**Figure 1:**
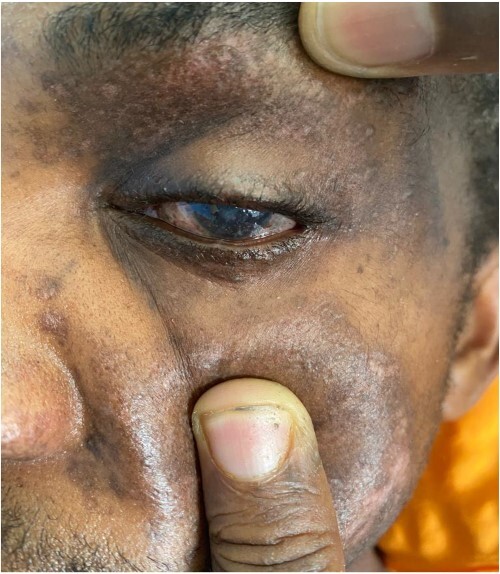
Conjunctival injury of the left eye of the patient, with multiple left periocular scars sustained from the blast injury.

**Figure 2:**
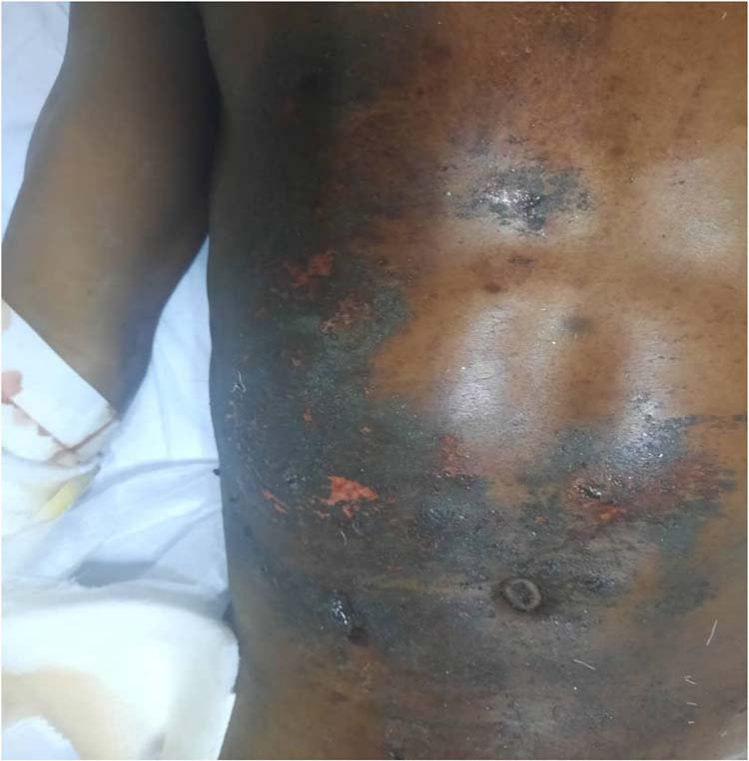
Presentation of multiple severe burn wounds on the anterior aspect of the abdomen and pelvis sustained following the blast injury.

**Figure 3:**
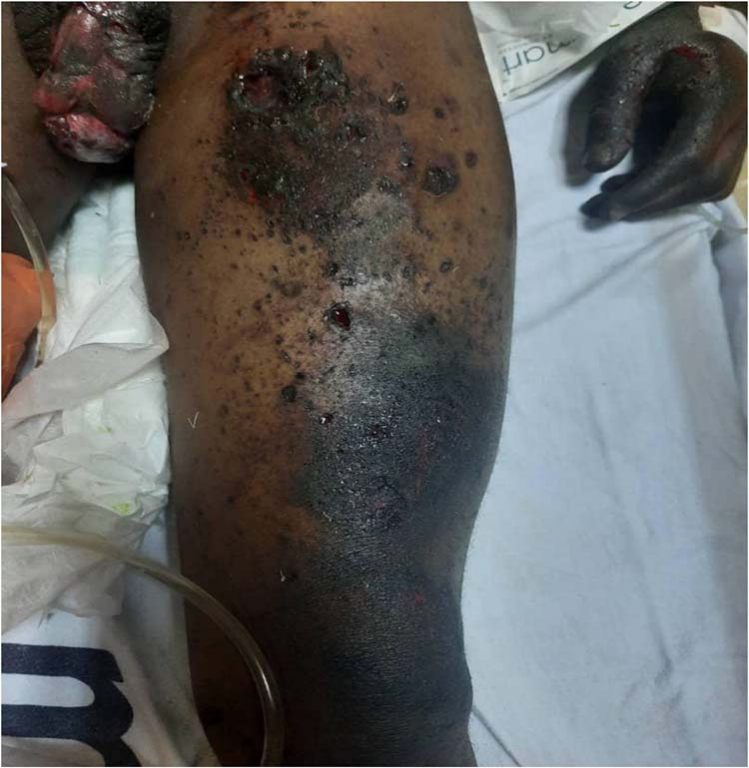
Rupture of the left hemiscrotum with exposed testis in the patient, with multiple deep burn wounds on the left thigh.

**Figure 4:**
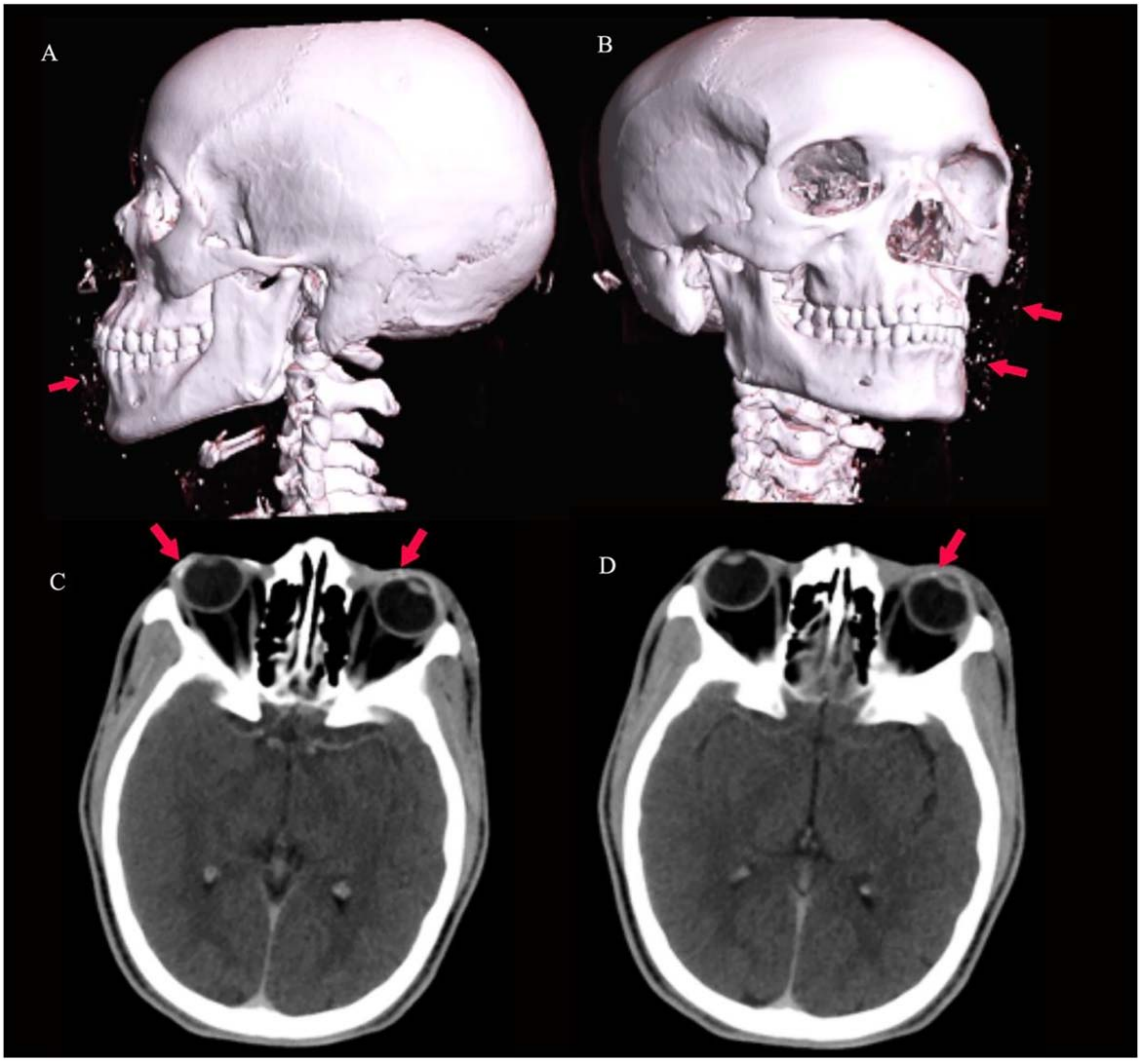
3D virtual reconstruction images (**A** and **B**) show multiple radio-opaque foreign bodies in the subcutaneous tissue of the face, as shown by arrows. Axial CT of the brain (**C** and **D**) at the level of the orbits shows conjunctival foreign bodies (shrapnel), as shown by arrows.

**Figure 5:**
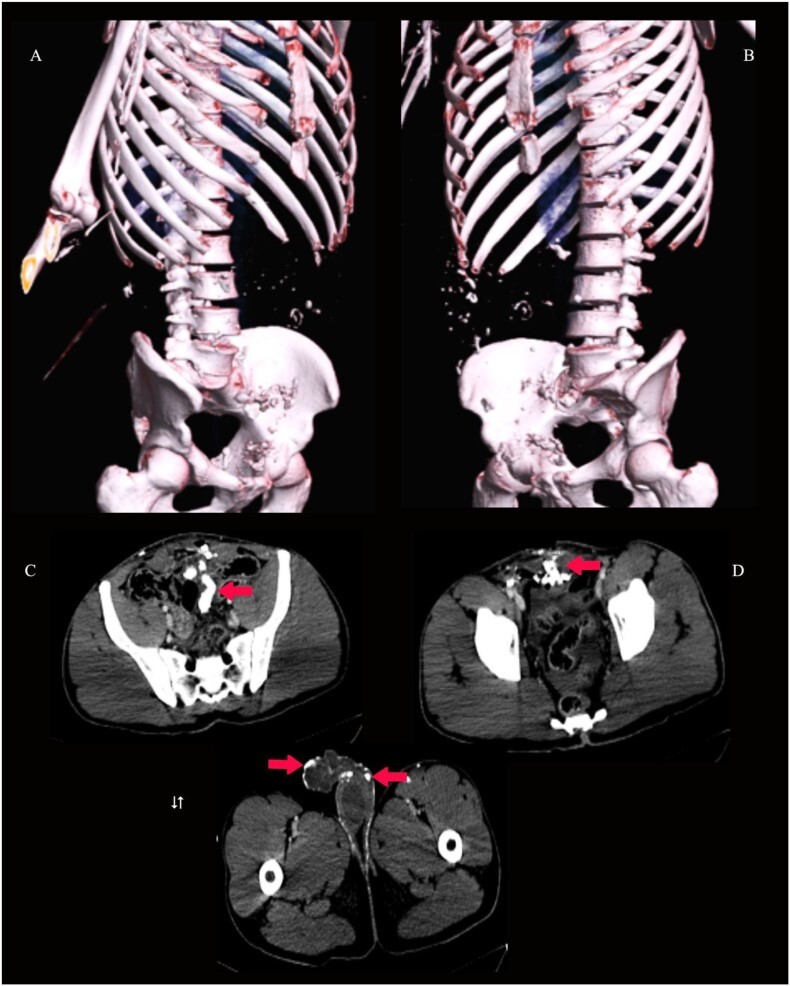
3D virtual reconstruction images (**A** and **B**) and axial CT pelvis (**C**, **D** and **E**) show innumerable radio-opaque foreign bodies (shrapnel) in the peritoneal cavity, anterior rectus abdominis muscle and the subcutaneous tissue of the penis, scrotum and left thigh, as shown by arrows.

A 27-year-old male patient who works in a mine was involved in a dynamite explosion. Following the incident, he suffered severe burn wounds to his right forearm, penis and scrotum, along with deep lacerations on his upper and lower limbs, the front of his chest and belly, and left eye. He claims that after the incident, he briefly lost consciousness. When he regained consciousness, he started experiencing constant headaches and impaired vision in his left eye. He denied having convulsions, vomiting, nausea, light-headedness or changes in bowel habits. He was neither pale nor cyanosed when he arrived at our emergency room, had a GCS of 15, was cognizant and was fully oriented. He was kept on oxygen support with NC. His vital signs on presentation were: 132/79 mmHg blood pressure, 84 bpm heart rate and 19 c/m respiration. He had multiple burn wounds, deep lacerations on the left side of his face, neck and right forearm with bone protrusion onto its dorsal surface, wounds on the anterior aspect of his chest and abdomen, a deep lacerated wound on his suprapubic aspect, and a lacerated and necrotic left testicle outside the hemiscrotum. Additionally, he had a laceration on the anterosuperior portion of his left thigh. He had a 30% TBSA and an overall MES of 5 (Figs 1, 2 and 3). He had a normally distended abdomen with multiple burn wounds and lacerations. It moved in unison with breathing, was only mildly tender to palpation and bowel sounds were noticed. However, there were no signs of peritonitis. His CVS and CNS examinations were rather unremarkable. From this point onwards, it was stipulated that the patient had suffered traumatic abdominal injuries, an open right forearm fracture, second-degree superficial burn wounds with a 30% TBSA and a left corneal injury. An X-ray of the chest revealed characteristics indicative of right middle lobe contusions. A CT scan revealed multiple sharp foreign bodies in the subcutaneous tissue of the left thigh and pelvis, anterior to the iliac artery and vein, the anterior abdominal wall perforating the rectus abdominis muscle, and the anterior chest wall, there was the presence of an exposed left testis and epididymis due to hemiscrotum rupture, sharp foreign objects were also found embedded in the conjunctiva of both eyes (Figs 4 and 5). The patient had preoperative examinations before being hurried into surgery. A 300-ml hemoperitoneum collection, an exposed innocent urinary bladder and a perforated transverse colon with minor faecal contamination were all observed during the surgery. The left testicle was lacerated, necrotic and exposed, and a traumatic circumcised penis and multiple wounds on the glans penis that involved the urethral mucosa were also observed. The procedures included an orchidectomy, a meatoplasty and a double-barrelled transverse colostomy. The patient had a good post-operative recovery. The wounds were dressed throughout the next days, adding to his extended ward stay. He was eventually discharged. A subsequent couple of weeks of follow-up care were unremarkable.

## DISCUSSION

Blast injuries can result in catastrophic physical, mental and psychological detriment to a patient. They have the potential to cause severe and life-threatening injuries. Healthcare systems now provide better patient care and can better manage casualties like bomb injuries. Nevertheless, the distinctive damage patterns these individuals typically come with make it difficult for physicians to diagnose and treat them. According to [2], high- (HE) and low-order explosives (LE) are the two categories of explosives. LE explosives produce a subsonic blast as opposed to an over-pressurized shock wave by HE. A person’s exposure to the aftereffects of the detonation depends on how close they are to the explosive agent. The magnitude of the overpressure felt increases with proximity [3]. When a person is subjected to primary blast injuries, gas-filled tissues such as the middle ear, lungs and gastrointestinal system are the most severely injured organs. Although secondary blast injuries are the most frequent explosion injuries, they can occur anywhere on the body and are caused by flying debris and fragments. The fragments might be non-metallic or metallic (shrapnel), as stipulated by [4]. Other injuries, such as ocular trauma, may lead to ocular tissue disruption, inflammation and infection [5].

Blast injuries should be managed with early life support measures emphasizing proper airway, breathing and blood circulation following the ATLS guidelines as they might potentially result in severe patient illness if not immediately assessed adequately. A thorough history taking and clinical examination follow once it has been determined that the patient is hemodynamically stable [6]. A regimen that includes steroid replacement, intensive blood sugar control, antimicrobial treatment and targeted intestinal decontamination is effective [7]. The ‘Trauma triad’ of coagulopathy, acidosis and hypothermia should also be assessed, then concurrent resuscitation strategies [8]. A physician should classify the risk of exposure to the blast injury to elicit appropriate responses from patients with blast injuries into penetrating injuries, mucous membrane exposures and superficial skin exposures without mucous membrane involvement [9]. Explosions may contain radiologic contamination; physicians should have protective gear when managing these patients. To guarantee tissue perfusion and prevent volume overload, fluid administration should be based on an evaluation of the central venous pressure and urine output. Additionally, based on clinical evaluations and radiological imaging modalities, patients should be assessed for intra-thoracic and intra-abdominal injuries. The recommended imaging would be a CT scan as it is highly sensitive in providing a detailed assessment of the viscera, retroperitoneum and abdominal wall, even if a chest X-ray is crucial as the initial imaging examination in trauma patients [10]. Regarding treatment methods, the more extensive the tissue damage, the more intricate the excision of the wound must be. It is essential to do a thorough surgical debridement of non-viable tissues, remove foreign bodies and leave an open, healthy wound. There is no need for serial debridement if the debridement is radical [10]. Testicular ruptures associated with blunt scrotal trauma, haematocele and haematoma formation command a surgical exploration and excision of necrotic tissues [11]. A damage control surgery is indicative of restoration of body physiology rather than anatomy as it involves rapid control of haemorrhage and contamination.

## CONCLUSION

Blast injuries are often affiliated with significant morbidity and mortality. They put a negative strain on the patient’s physical and emotional health. These injuries should be evaluated following ATLS recommendations for the early care of trauma patients because, if ignored, they might have severe life-threatening consequences. It is essential for physicians to completely comprehend the pathophysiological characteristics of blast injuries in order to manage complicated injury presentations that their patients may present.
